# CHANCE: comprehensive software for quality control and validation of ChIP-seq data

**DOI:** 10.1186/gb-2012-13-10-r98

**Published:** 2012-10-15

**Authors:** Aaron Diaz, Abhinav Nellore, Jun S Song

**Affiliations:** 1 Institute for Human Genetics, UCSF, 513 Parnassus Avenue, Box 0794, San Francisco, CA 94143-0794, USA; 2 The Eli and Edythe Broad Center of Regeneration Medicine and Stem Cell Research, UCSF, 35 Medical Center Way, San Francisco, CA 94143-0525, USA; 3 Department of Epidemiology and Biostatistics, UCSF, 185 Berry Street, Lobby 5, Suite 5700, San Francisco, CA 94107, USA; 4 Department of Bioengineering and Therapeutic Sciences, UCSF, 513 Parnassus Avenue, San Francisco, CA 94143-0912, USA

## Abstract

ChIP-seq is a powerful method for obtaining genome-wide maps of protein-DNA interactions and epigenetic modifications. CHANCE (CHip-seq ANalytics and Confidence Estimation) is a standalone package for ChIP-seq quality control and protocol optimization. Our user-friendly graphical software quickly estimates the strength and quality of immunoprecipitations, identifies biases, compares the user's data with ENCODE's large collection of published datasets, performs multi-sample normalization, checks against quantitative PCR-validated control regions, and produces informative graphical reports. CHANCE is available at https://github.com/songlab/chance.

## Rationale

The foremost question that challenges an experimentalist about their ChIP-seq is, 'did my experiment work?' CHANCE (CHip-seq ANalytics and Confidence Estimation) is a software package that provides quantitatively rigorous yet intuitive answers to this fundamental question in the following ways:

1. CHANCE assesses the strength of immunoprecipitation (IP) enrichment to identify potentially failed experiments. CHANCE identifies insufficient sequencing depth, PCR amplification bias in library preparation, and batch effects.

2. CHANCE identifies biases in sequence content and quality, as well as cell-type and laboratory-dependent biases in read density. Read-density bias reduces the statistical power to distinguish subtle but real enrichment from background noise [1-3]. CHANCE visualizes base-call quality and nucleotide frequency with heat maps. Furthermore, efficient techniques borrowed from signal processing uncover biases in read density caused by sonication, chemical digestion, and library preparation.

3. CHANCE cross-validates enrichment with previous ChIP-qPCR results. Experimentalists frequently use ChIP-qPCR to check the enrichment of positive control regions and the background level of negative control regions in their immunoprecipitation DNA (IP) relative to input DNA (Input). It is thus important to verify whether those select regions originally checked with PCR are captured correctly in the sequencing data. CHANCE's spot-validation tool provides a fast way to perform this verification. CHANCE also compares enrichment in the user's experiment with enrichment in a large collection of experiments from public ChIP-seq databases.

Despite having different goals, some software packages partially overlap with CHANCE in functionality: htSeqTools [4] is an R package with routines for coverage estimation, peak calling, and downstream analysis of ChIP-seq data. Interestingly, its use of Lorenz curves to estimate sample coverage is similar in mathematical principle to the signal-to-noise ratios previously used by us and others to construct estimates of the size and quality of the background fraction of IP [1,2]. By contrast, CHANCE provides statistics on coverage, as well as percentage enrichment for signal and multi-sample scaling. Other software visualizes the distribution of quality scores and base calls that may be useful in choosing parameters for mapping reads to a reference genome [5-8]. Some programs can also trim and filter reads based on base-call quality metrics [9-12]. These programs nevertheless do not address biases in read density that can affect the reliability of called peaks and do not estimate the strength of IP enrichment. CHANCE not only incorporates the functionality of other software, but also has novel features that can significantly facilitate the quality control step of ChIP-seq analysis.

While Python scripts and Java applications are available for correcting read density for mappability and GC content biases [3], to our knowledge, no publicly available software today identifies biases that may arise due to sonication, chemical digestion, or laboratory-specific protocols. None of the aforementioned software has more than 1/4 of CHANCE's features (see the feature comparison table in Additional file 1). Of the ten software packages compared, seven require programming knowledge, and three are sequencing platform specific. In contrast, CHANCE has an intuitive graphical interface and works with reads from any platform. CHANCE runs on Windows, Mac OS, and Linux and does not require any programming or knowledge of statistics. It is a comprehensive, statistically rigorous application: it provides a bird's-eye view of the quality of a ChIP-seq data set, it allows experimentalists to compute multiple quality metrics, and it generates informative images as output graphical reports and figures. Only CHANCE provides a comprehensive suite of ChIP-seq quality controls in a user-friendly graphical interface.

## Results

### Data sets CHANCE can analyze

CHANCE works with reads mapped to a reference genome from IP and control (Input) samples. It can import reads in BED, tagAlign [13], SAM, and BAM [14] formats, as well as BOWTIE [15] output. Its interactive plots include a suite of plotting tools and an export utility to produce informative graphics in most standard formats. In addition to interactive plots, CHANCE also generates a text log of the session containing a summary of the statistical tests performed.

### Estimating the strength of IP enrichment

IP enrichment strength is important for calling robust peaks that correspond to transcription factor (TF) binding sites or epigenetic modification sites. To estimate the IP strength, CHANCE attempts to decompose the population of IP reads into two distinct components: those pulled down by the antibody, and background. To accomplish this task, CHANCE uses signal extraction scaling (SES), which is based on order statistics [1]. SES estimates the percentage of the IP data enriched for biological signal, the coverage of IP reads corresponding to DNA fragments pulled down by the antibody, and a scaling factor for properly normalizing IP and Input together. The level of IP enrichment can be used to classify whether an experiment was successful. We have trained CHANCE on thousands of ChIP-seq samples derived from the ENCODE repository (see Materials and methods). CHANCE reports a *q*-value for the IP enrichment level based on this training data and uses the *q*-value to identify potentially failed experiments.

**Figure 1:**
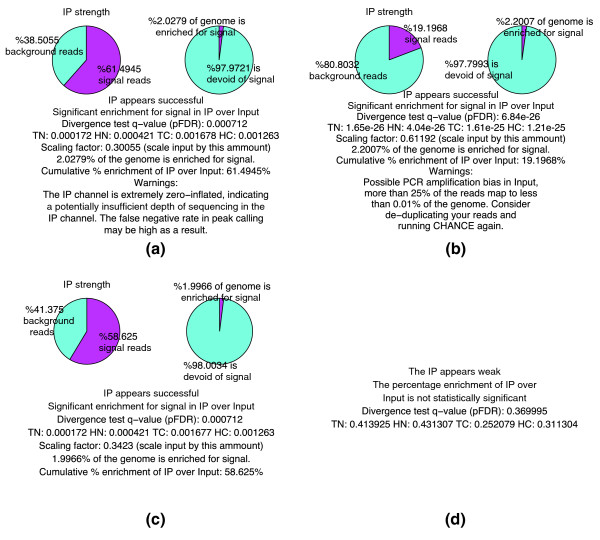
**Statistical summary of IP enrichment strength**. Each panel shows a summary statement of IP strength produced by CHANCE for a different sample. For each sample, CHANCE summary statements include: the statistical significance of IP enrichment, or the lack thereof; pie charts estimating the percentage of reads corresponding to DNA fragments pulled down by the antibody and the percentage of the genome enriched for biological signal; and, warning statements of possible bias or lack of sequencing depth. **(a) **The analysis results for H3K4me3 in human embryonic stem cells (HESCs; GEO GSM727572). Although this sample shows significant enrichment for signal, it also displays a possible lack of sufficient sequencing depth, which will result in a high false negative rate in peak detection. **(b) **The analysis results for H3K4me3 in mouse neural stem cells (NSCs). This sample shows decent enrichment, but CHANCE also detects an amplification bias in the input channel and alerts the user. **(c) **The results for the same sample as in (b) after bioinformatic de-duplication of reads. De-duplication has suppressed the amplification bias, recovering biological signal in the IP. The warning message has disappeared after de-duplication. **(d) **The summary statement for *CARM1 *in HESCs (GEO GSM801064). For this sample, the IP appeared extremely weak; CHANCE is unable to produce pie chart enrichment estimates as in the previous samples, but it nevertheless reports the false discovery rate (FDR) associated with the test for enrichment. There are four false discovery rates reported, each estimated on a separate subset of training data. Their abbreviations are as follows, HC: histone mark - cancer tissue; HN: histone mark - normal tissue; TC: transcription factor binding site - cancer tissue; TN: transcription factor binding site - normal tissue.

**Figure 2:**
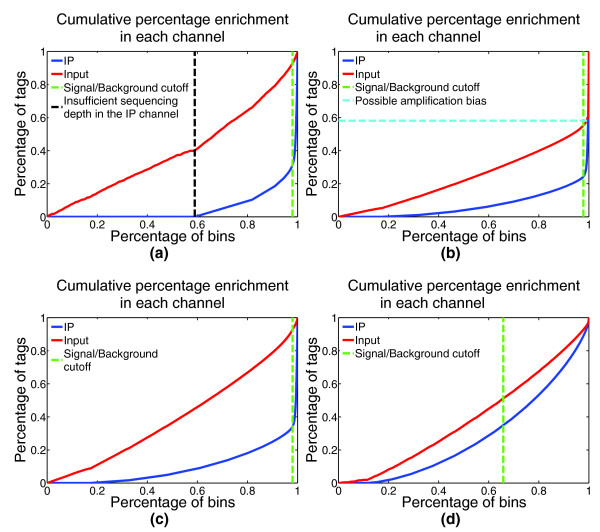
**Graphical summary of IP enrichment**. In addition to summary statements, CHANCE produces graphical visualizations of IP strength by separating background regions from ChIP-enriched regions. For a complete discussion on the statistical interpretation of these plots, see [1]. Briefly, points on the *x*-axis correspond to percentages of the genome, and points on the *y*-axis correspond to percentages of the total number of reads. The point at which the distance between the IP and Input percentages is maximized is denoted by the green line; the greater the separation between IP and Input at this point, the better the IP enrichment. The shapes of the two curves also provide useful information about the data. **(a) **The IP curve for H3K4me3 in human embryonic stem cells (HESCs; GEO GSM727572) stays near 0 until it reaches 0.6, indicating that 60% of the genome did not have sufficient coverage in the IP channel. CHANCE detects this insufficient sequencing depth and indicates the percentage of uncovered genome by a black line. **(b) **For H3K4me3 in mouse neural stem cells (NSCs), CHANCE indicates amplification bias with a turquoise line, identifying over 60% of the reads mapping to a small percentage of the genome. **(c) **The same sample as in (b) is shown after de-duplication. CHANCE does not detect any amplification bias after de-duplication. **(d) **This figure exemplifies a weak IP (*CARM1 *in HESCs; GEO GSM801064), where the IP and Input curves are not well separated.

In addition to assessing the strength of IP, it is also important to monitor the levels and sources of different biases present in the data. Identifying these biases is useful for optimizing experimental protocols. During the estimation of IP strength, CHANCE thus also detects several forms of bias. Figure 1 shows typical summary statements, and Figure 2 shows graphical representations of IP strength estimation for several samples, produced by CHANCE. Figures 1a,b and 2a,b are samples with strong ChIP enrichment, but also with substantial biases; Figures 1c and 2c show a successful low bias ChIP; and, Figures 1d and 2d correspond to a very weak ChIP. Figures 1a and 2a show the CHANCE outputs for a H3K4me3 ChIP-seq in human embryonic stem cells (Gene Expression Omnibus (GEO) accession GSM727572). Although CHANCE finds significant enrichment in IP relative to Input, it also detects that almost 60% of the genome has zero coverage, indicating insufficient sequencing depth in the IP. Figures 1b,c and 2b,c show results for a H3K4me3 ChIP-seq in neural stem cells (NSCs) from the murine sub-ventricular zone obtained from the Lim lab at University of California, San Francisco (UCSF) [1]. In Figures 1b and 2b, CHANCE finds significant enrichment in the IP, but it also detects a significant bias in the Input channel - that is, it is found that almost 40% of the reads map to less than 0.001% of the genome. In this data set, the average read density is about 10 reads/kbp; however, for less than 0.001% of the genome, the read density reaches over 50,000 reads/kbp. This kind of outlier coverage often indicates a large number of duplicate reads, which can arise from PCR amplification bias during library preparation [16]. Indeed, after de-duplicating the set of reads and re-running CHANCE, we see a greater fraction of reads corresponding to biological signal, as shown in Figures 1c and 2c. In Figures 1d and 2d, we show an example of a ChIP-seq experiment for *CARM1 *in human embryonic stem cells (GEO accession GSM801064), where the IP sample is statistically indistinguishable from Input.

**Figure 3:**
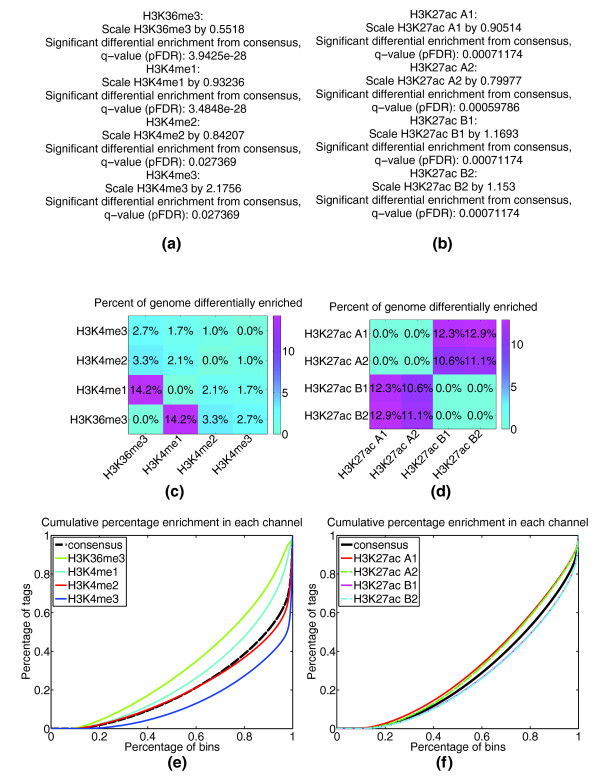
**Multi-IP normalization**. This figure shows CHANCE output for the multi-IP normalization module. **(a-d) **CHANCE produces a summary statement (a-b), a pairwise sample differential enrichment matrix (c-d), and a graphical representation of the normalization process. The graphical representation gives the same type of plot as in IP strength estimation for each IP sample, as well as the consensus of the IP samples; see Materials and methods. The summary statement quantifies the graphical representation by giving the statistical significance of the difference of each sample from the consensus. The differential enrichment matrix computes the percentage of the genome differentially enriched between all pairs of samples, using the same technique for IP-Input comparison used in IP strength estimation; see Materials and methods. **(a,c,e) **Multi-IP normalization of H3K4me1, H3K4me2, H3K4me3, and H3K36me3 in human embryonic stem cells (H1 HESCs), from the Broad ENCODE data. **(b,d,f) **The capacity of CHANCE multi-IP normalization to detect batch effects. The clustering of technical replicates (denoted by 1 and 2) for each biological replicate (denoted by A and B) seen in (f) is quantified in the pairwise differential enrichment matrix (d), which shows a statistically insignificant percentage of the genome differentially enriched between replicates but a non-negligible percentage of the genome differentially enriched between batches.

CHANCE can also compare two or more IP samples (for example, samples obtained before and after knocking down a protein of interest) by constructing a consensus profile based on signal processing techniques designed to identify regions of mutual enrichment [17-19] (see Materials and methods). The samples are then normalized to the consensus using SES, and the statistics on sample pairwise differential enrichment as well as scaling factors for multi-sample comparison are reported. Figure 3 gives an example of CHANCE output for multi-IP comparison. Figure 3a,c,e demonstrates such an analysis by comparing H3K4me1, H3K4me2, H3K4me3, and H3K36me3 in human embryonic stem cells (H1 HESCs), from Broad Institute tracks available in the ENCODE repository. Figure 3a is a summary statement of the statistical significance of the difference between a given sample and the consensus; Figure 3c provides a pairwise estimate of the fraction of the genome differentially enriched for a given sample; and Figure 3e gives a graphical representation of the multi-IP comparison.

It is well known that sending samples to a sequencing facility at different times can result in unwanted batch effects. To facilitate the detection of such variability, CHANCE automatically identifies potential batch effects in replicate data. For example, Figure 3b,d,f shows a four-sample normalization of two batches (A and B) and two technical replicates (rep1 and rep2) for H3K27ac in murine whole limb from the Ahituv lab at UCSF (data not published). The batch effect can be seen in graphical form in Figure 3f, where batch A and batch B appear to cluster together. In Figure 3d, the batch effect is further quantified by the estimates for the percentage of the genome differentially enriched amongst the four samples. In particular, in Figure 3d, CHANCE was unable to detect statistically significant differential enrichment between technical replicates; by contrast, it found 10 to 12% of the genome to be differentially enriched between the samples from different batches, suggesting a non-negligible batch effect between A and B. CHANCE thus provides a powerful tool to aid scientists in optimizing their ChIP and library construction protocols by identifying biases and estimating the relative effectiveness of different methods.

### Detecting bias in the library preparation and sequencing

ChIP-seq data may have many biases and artifacts that can significantly influence the interpretation of the data. CHANCE can rapidly assess the quality of ChIP-seq by detecting two types of bias: bias in base-call content and quality and bias in read density. Severe bias in base-call content and quality can indicate problems with the sequencing [7]. Moreover, the genome-wide distribution of reads is never uniform. Biases in read density for Input have been shown to occur at transcription start sites and internal gene exon boundaries [3] and can also be observed in a cell type-dependent fashion [1]. In addition to the aforementioned ability to detect PCR amplification bias, CHANCE provides several tools to analyze the sources of bias more completely, as described below.

#### Analyzing nucleotide content and base-call quality

**Figure 4:**
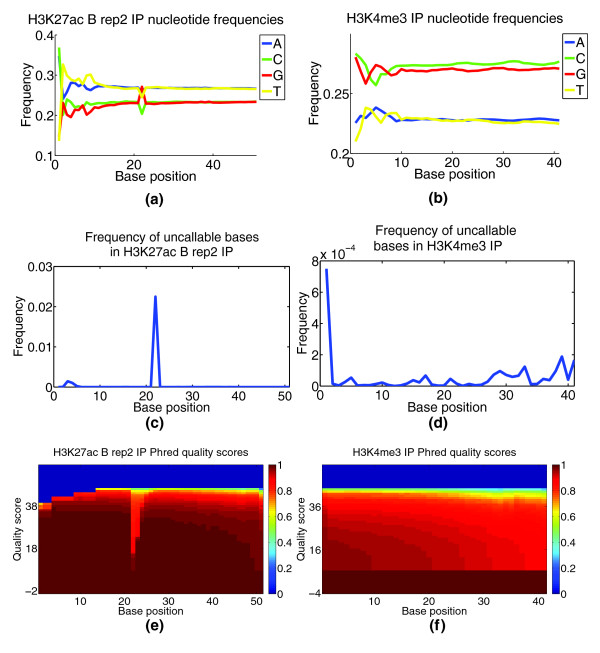
**Base call content and quality bias**. This figure demonstrates CHANCE output for base call content and quality bias module. **(a,b) **Plot of nucleotide frequency as a function of base position. **(c,d) **Plot of the frequency of uncallable bases as a function of base position. **(e,f) **Heat maps of Phred quality scores, where the *x*-axis corresponds to base position and the *y*-axis to Phred quality score. The color of a given (*x,y*) pair gives 1 minus the cumulative density of reads with a quality score of *y *or less. That is, it gives the fraction of reads with a quality score of *y *or more; so, the redder, the better. (a,c,e) H3K27ac IP in mouse whole limb from the Ahituv lab at UCSF. These samples show a marked drop in quality, a rise in uncallable bases, and an abrupt change in nucleotide frequencies for positions 22 to 24, indicating potential problems with the sequencing. For comparison, (b,d,f) show results for H3K4me3 IP from mouse NSCs from the Lim lab at UCSF, which exhibit relatively low bias.

CHANCE displays nucleotide frequency plots as well as the frequency of uncallable bases. It shows the distribution of Phred quality scores at each base. A stretch of uncallable bases, or a stretch of bases with unusual nucleotide content or unusually low base-call quality scores can indicate problems with the sequencing. In Figure 4, we compare the frequency of uncallable bases and nucleotide content at each sequenced base location between the sub-ventricular zone NSC H3K4me3 and whole-limb H3K27ac data sets. We see in the H3K27ac data a stretch of bases from positions 22 to 24 with a noticeable GC content bias and a high frequency of uncallable bases. Moreover, we see a dip in base-call quality scores over the same stretch of bases. This kind of analysis can provide quick, valuable feedback to the sequencing facility.

#### Detecting library preparation bias

**Figure 5:**
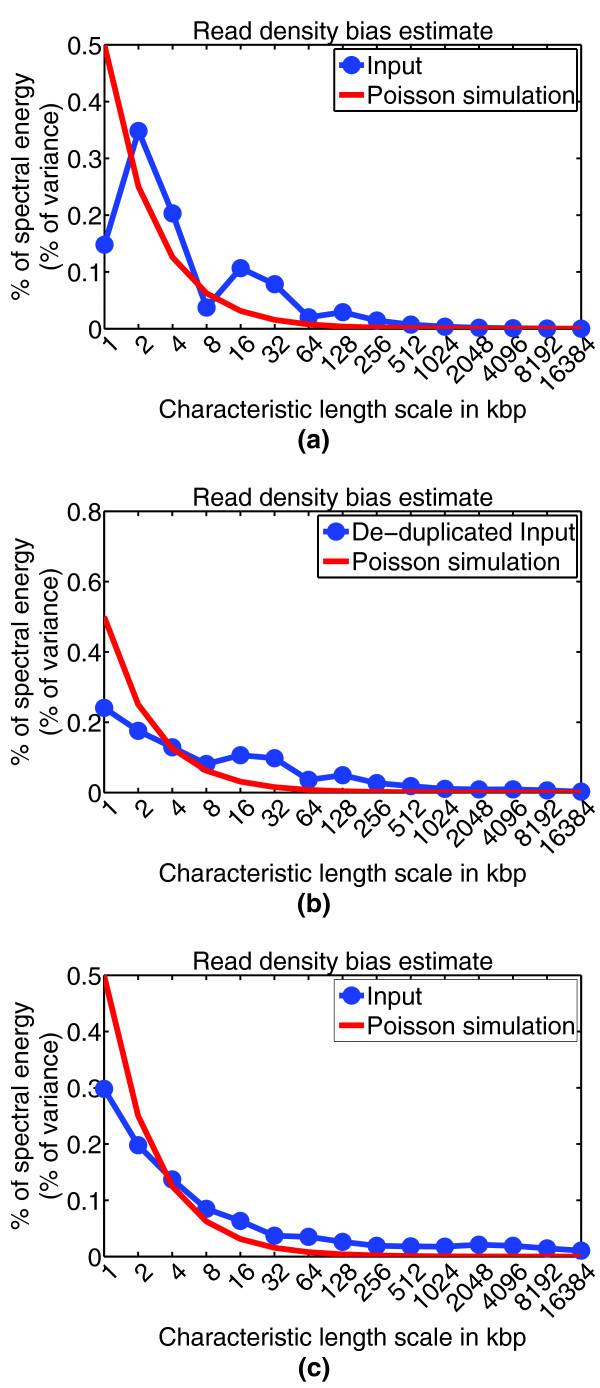
**Read density bias**. This figure shows CHANCE output for the read density bias estimation module. The *x*-axis denotes length scale, from 1 kbp to 16.384 Mbp. The *y*-axis denotes percentage of variance in data at a given length scale (spectral energy). The blue curve is the user's data, while the red curve denotes the distribution of an idealized bias-free data set generated by a Poisson simulation (see Materials and methods). Noticeable deviation of the blue curve from the red simulation curve might indicate copy number alterations in cancer cells, amplification bias in generating duplicate reads, GC content and mappability bias, or inability to sonicate heterochromatin regions. **(a) **Mouse NSC Input from the Lim lab with significant IP enrichment, but also with a heavy amplification bias in the input channel. **(b) **After de-duplicating reads, the sample in (a) shows a reduction in bias as demonstrated by a better agreement between the user's data and the Poisson simulation (compare Figures 1b,c and 2b,c). **(c) **For comparison, this panel shows mouse whole limb Input from the Ahituv lab, which demonstrates relatively low bias in read density.

Bias in Input read density might reflect copy number alterations in cancer cells, amplification bias in generating duplicate reads, GC content and mappability bias, or inability to sonicate heterochromatin regions. These biases occur at different genomic length scales, and it can be useful to assess the characteristic length scales at which major biases occur, such as to obtain a rough picture of amplified fragment sizes in cancer cells. CHANCE detects bias in read density by using a signal processing technique known as spectral analysis. This technique decomposes the variation in read density into variations on a set of characteristic length scales. CHANCE then compares this decomposition to idealized data, Poisson-simulated at the same depth and coverage as the user's provided data set. Figure 5 shows a spectral analysis of the Input channels of the mouse sub-ventricular zone and whole limb data sets. On the *x*-axis is a set of length scales, from 1 kbp to 16.384 Mbp. On the *y*-axis is the percentage of variance in read density observed in the user's data at each length scale. If the chromatin sonication or digestion process were unbiased - or, if the library preparation, sequencing, and mapping were all done without bias or error - then the break points introduced in chromatin would be uniformly distributed genome-wide, and the number of reads mapping to a particular region would be approximately Poisson-distributed with a mean constant throughout the genome. This expected trend would appear in the spectral analysis plots (Figure 5) as a spectral energy distribution that was highest at 1 kbp, indicating a read density profile composed primarily of high frequency fluctuations about a global mean. The spectral energy distribution would then rapidly drop down as we increase the length scale along the *x*-axis. Figure 5a,b shows Input from mouse NSCs both before and after de-duplication (compare Figure 1b,c and Figure 2b,c). Note that the distribution more closely matches the ideal simulated data after de-duplication, indicating a decrease in bias. For comparison, Figure 5c demonstrates relatively low read density bias in the Input data from mouse whole limb.

### Performing validation and comparison to known data sets

Spot validation of ChIP-seq peaks at sites known *a priori *to be enriched can provide additional confirmation of the success of an experiment. Comparison with other experiments of the same type can also help assess the relative quality of the user's data. These tests provide additional evidence that a ChIP-seq data set is reliable, as described below.

#### Validating ChIP enrichment on a candidate list of regions

**Figure 6:**
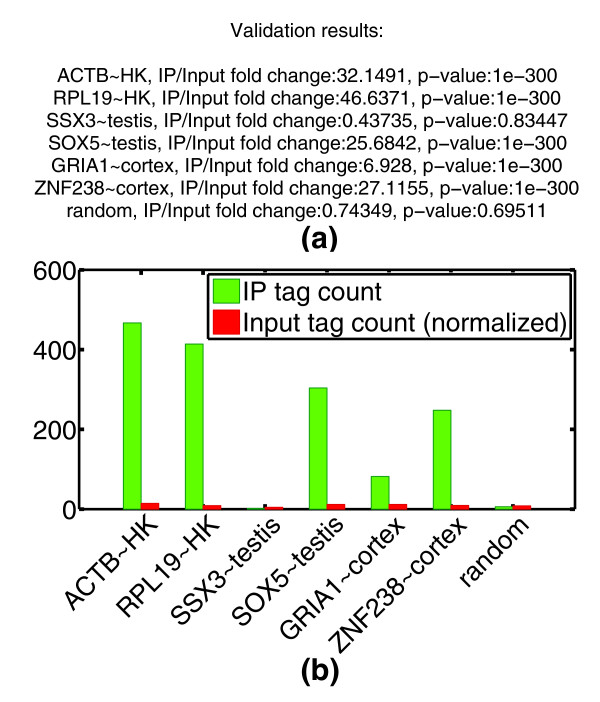
**Spot validation**. This figure demonstrates CHANCE's spot validation module. H3K4me3 in H1 HESCs from the Broad ENCODE data is spot validated for promoter regions of known housekeeping and tissue-specific genes. **(a) **The summary statement gives the IP over Input fold-change in read count as well as a *P*-value based on a Poisson null model (see Materials and methods). A random locus is added for comparison. The putative proximal promoter was estimated to 3 kbp upstream to 3 kbp downstream of the transcription start site. **(b) **Graphical representation of the results. On the *x*-axis, we have the gene symbols followed by the tissue type with which their expression is commonly identified; HK denotes 'house keeping' or ubiquitously expressed genes. The *y*-axis shows the number of reads mapping to the corresponding promoter region, both in IP and Input.

CHANCE allows the user to enter a list of candidate regions for spot validation. For example, experimentalists typically check positive control regions via ChIP-qPCR and would be interested in checking the enrichment of those regions in their ChIP-seq data. The spot-check routine returns the fold-change of IP over Input and an estimate of its statistical significance at each of the user-defined locations. Figure 6 shows an example of spot validation of the H3K4me3 H1 HESC data set.

#### Comparing user data to other experiments

**Figure 7:**
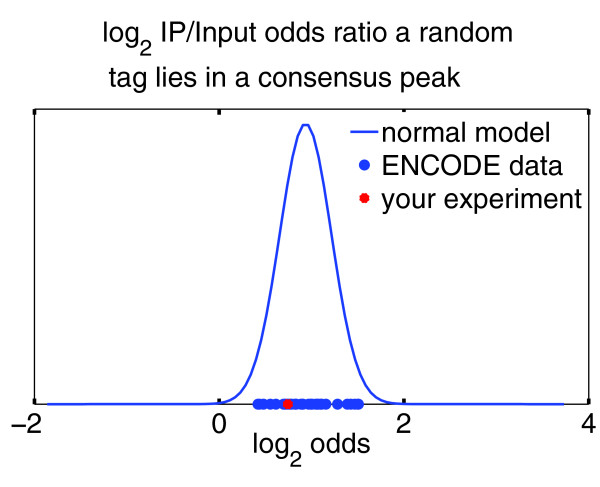
**Comparison with ENCODE**. CHANCE can quickly compare the user's experiment with thousands of other experiments in the ENCODE repository. This figure shows CHANCE's output for H3K27me3 ENCODE data from the Broad Institute. On the *x*-axis is the IP over Input odds ratio that a randomly chosen read from the user's sample will lie in the union of all peaks of all ENCODE samples for the same transcription factor or epigenetic mark as the user's ChIP. The blue bell curve is a probabilistic model fitted to all available data. The blue circles denote all available data sets from ENCODE, and the red star is the user's sample. Intuitively, the user's sample has a poor overlap with the ENCODE data if the red star lies in the extreme left tail. The figure shows that the H3K27me3 sample is not an outlier when compared to other ENCODE samples.

One useful way of checking whether a ChIP-seq experiment was successful is to compare its peak list with those obtained by other scientists in other cell types. The overlap will not be perfect, but a very poor overlap will suggest that the experiment might not have worked. To facilitate this process, CHANCE compares the user's data to other data sets of the same ChIP type in the ENCODE repository. However, CHANCE does not detect peaks to carry out this comparison, but it rather compares the genome-wide enrichment profile of the user's raw data to all available ENCODE ChIP-seq data for the same TF or epigenetic mark. It then uses these ENCODE data sets to compute the probability that the user's experiment is a statistical outlier. Although agreement with ENCODE data does not guarantee an experiment was successful, a high probability of being an outlier may indicate a data set has problems. Figure 7 shows an example of CHANCE comparison to known data sets for the H3K27me3 in H1 HESCs, also from Broad ENCODE data sets.

## Discussion

**Figure 8:**
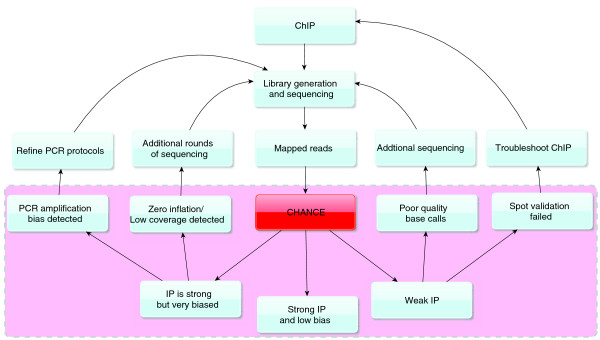
**A typical workflow with CHANCE**. CHANCE can provide a scientist with feedback regarding the success of their experiment, as well as how their protocols may be improved. CHANCE is designed to work with reads mapped to a reference genome. The dotted pink region illustrates the useful quality metrics computed by CHANCE to assess a ChIP-seq experiment. One can use these intuitive results to determine if the experiment is satisfactory or if additional protocol modification or sequencing is required.

Although software exists for read-trimming and filtering prior to mapping reads to a reference genome and for downstream analysis such as peak calling, there is still a need for a software package designed specifically for ChIP-seq quality control that can provide immediate feedback to experimentalists. Moreover, as ChIP-seq becomes more widely used, there is a need for tools that do not require programming skills to use and that can produce high quality graphical reports. CHANCE fills that gap. Figure 8 illustrates how CHANCE might fit into a typical work flow. CHANCE takes mapped reads in commonly used formats and outputs useful statistical summaries (for example, those shown in the pink shaded region in Figure 8), which can then provide immediate feedback to the experimentalist and sequencing facility. By serving as a key link between data generation and downstream analysis, CHANCE will help expedite the analysis and optimization of ChIP-seq experiments and will help maintain the high quality requisite for better reproducibility and consistency.

## Materials and methods

### IP enrichment estimation

CHANCE uses SES [1] to compute the largest subset of the genome for which the distribution of reads in IP matches that in Input. This procedure partitions the genome into two sub-regions: a region of potential biological signal and a background region. A scaling factor for IP-Input normalization can then be computed by mean normalizing the read density in IP background to the read density, in the same region, from the Input channel. As a byproduct of this process, an estimate of differential enrichment in the IP over Input (the percentage increase in mean tag density in IP compared to Input), as well as an estimate of the percentage of the genome enriched for signal (the relative size of the non-background region) can be obtained. As described in [1], we use a divergence test on the percentage allocation of reads in each channel to determine a *P*-value for statistical significance.

In order to ascertain the precision and recall of the divergence test as a classifier of successful experiments, we calibrated CHANCE on a data set obtained from the ENCODE repository. We downloaded all ENCODE ChIP-seq data sets with replicate inputs (Additional file 2). We then re-sampled from the genomic distribution of reads in each dataset ten times; these re-sampled data were used to produce an empirical distribution of divergence statistic from all possible cell type-matched IP-Input or replicate Input-Input pairs. The divergence test statistic and associated *P*-value were calculated for each pair. The positive tests derived from IP-Input comparisons were taken as true positives, and the positive tests for Input-Input comparisons were assumed false positives. This is reasonable under the assumption that the ENCODE repository is curated and the vast majority of IP-Input pairs represent successful experiments, while the vast majority of comparisons between Input replicates should show no differential enrichment. In this fashion, we estimate a *q*-value (positive false discovery rate) for a given value of the divergence test statistic as the fraction of Input-Input pairs in the set all samples with divergence test values greater than or equal to the user's divergence test value. The *q*-value is thus interpreted as the fraction of comparisons from ENCODE that show differential enrichment at the level of the user's data, but turn out to be technical replicates of the Input channel.

**Figure 9:**
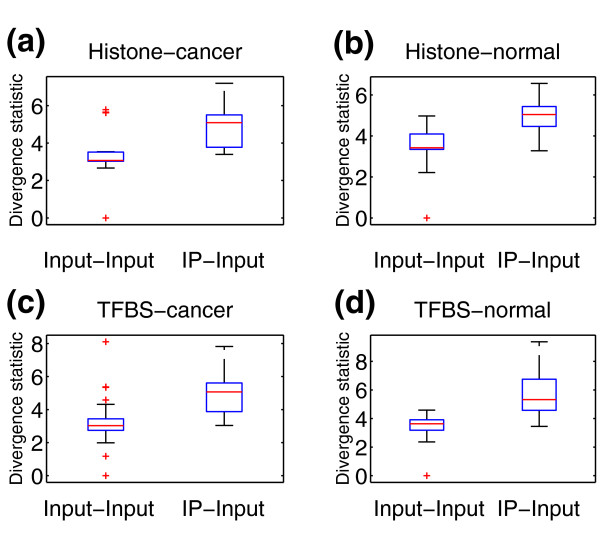
**The divergence test statistic by sample type**. These box plots show the distributions of the divergence test statistic for IP-Input and Input-Input comparisons for the ENCODE training data grouped into sample types: **(a) **histone mark in cancer cells; **(b) **histone mark in normal cells; **(c) **TF in cancer cells; and **(d) **TF in normal cells. The distributions of the divergence test statistic have slightly stronger mixing for histone data (a,b) compared to TF data (c,d), as evidenced by the higher whiskers in the TF IP-Input box plots. Moreover, the Input-Input comparisons for cancer samples (a,c) show a greater number of outliers denoted by red plus signs with large divergence test values compared to normal samples (b,d). To account for these differences, CHANCE estimates false discovery rates separately for each sample type. TFBS, transcription factor binding site.

While the majority of histone mark ChIP-seq enrichment profiles tend to be spread out, profiles for TFs tend to be more punctate. This spreading can result in a reduction in ChIP signal and lead to a stronger mixing between the distributions of Input versus Input and IP versus Input divergence test statistics for histone mark ChIP-seq, whereas the distributions are more separable for TF ChIP-seq, as shown in Figure 9. This bias might increase the *q*-value estimate for histone ChIP-seq. Furthermore, cancer cells frequently suffer from genomic instability, and copy number alterations in background regions can artificially increase the local read density. This bias is reflected in the fact that the null distribution of divergence test values for Input versus Input comparisons has a heavier tail in cancer samples compared to normal tissues. In order to account for these potential biases, CHANCE reports *q*-value estimates of enrichment separately for each of the following categories of training data: (a) histone mark in normal cells, (b) histone mark in cancer cells, (c) TF in normal cells, (d) TF in cancer cells, and (e) all samples. Figure 9 shows whisker-box plots of the divergence test statistic for the ENCODE training data separated into the above categories. We do indeed see a slightly stronger mixing of the IP-Input with the Input-Input distribution when comparing histone mark data in the top panels (Figure 9a,b) with the TF data in the bottom panels (Figure 9c,d), as evidenced by a higher top whisker mark in the IP-Input distributions for the TF data compared to the histone data. Moreover, comparing the cancer data in the left panels (Figure 9a,c) with the normal data on the right panels (Figure 9b,d) shows that the cancer data distribution of Input-Input comparisons is indeed heavier tailed than the Input-Input distribution of the normal data. Consequently, one should expect higher false discovery rates in histone and cancer samples for the reasons mentioned previously. CHANCE will alert the user to a possibly failed sample if all of the *q*-values are above 5%, but the user may also compare their experiment by category.

### Detection of insufficient sequencing depth in the Input channel

As in [1], let *p*(*α*) denote the percentage of reads in the IP channel contained in the first α percent of 1 kb non-overlapping bins sorted in an increasing order of read density. Similarly, let *q*(*α*) denote the percentage of the matching tag counts in Input, reordered by the sorting induced by the sorting of the IP channel. If IP had sufficient enrichment, then we must have *p*(*α*) ≤ *q*(*α*), since reads accumulate significantly in a small genomic subset targeted by IP, while the majority of sequences in the Input channel are more uniformly distributed throughout the genome. On the other hand, if there is insufficient sequencing depth in the Input channel, then there will be abundant zero counts in Input tag bins; and for α sufficiently small, we will have *q*(*α*) ≤ *p*(*α*). If CHANCE detects this crossing of *p*(*α*) from below by *q*(*α*), it reports a warning of potential low coverage in the Input channel.

### Detection of insufficient sequencing depth in the IP channel

Similarly, if there is insufficient sequencing depth in the IP channel, there will likewise be abundant zero counts in its tag bins. This implies that *p*(*α*) will be zero for *α *≤ *α*_0 _for some *α*_0 _> 0, *α*_0 _therefore being the percentage of the genome with zero coverage. In some extreme cases, the maximal percentage differential enrichment of IP over Input occurs at *α*_0 _(for example, Figure 2a), indicating that an insufficient coverage in the IP channel can create too many zero-count bins, which drive the background noise estimate to zero. In this case, CHANCE will excise the regions of zero coverage in the IP and re-compute the percentage enrichment; it will also report a warning of insufficient sequencing depth in the IP channel.

### Detection of potential PCR amplification bias

If 25% or more of the reads from either channel map to less than 1% of the genome, then there tend to be severe point spikes in the enrichment profile, most likely corresponding to mapping or PCR biases. CHANCE reports a warning if this condition is satisfied.

### Read density bias estimation

The read density bias estimation module has two components: a spectral analysis and an idealized Poisson simulation based on the user's data. Spectral analysis is a tool that allows one to determine how much of the variance in local coverage in the Input channel occurs over a given genomic length scale. An ideal Input sample would have only small fluctuations in coverage as we move along the genome and would have all of its variance at small length scales. In a more realistic setting, the distribution of variance would be concentrated at a small length scale and rapidly decrease as a function of increasing length scale, displaying some minor long-distance correlations in read density. A heavily biased sample will have systematic and reproducible fluctuations in mapped read density at several length scales, corresponding to condensed chromatin fragments resistant to sonication, PCR amplification bias, or genomic amplification and deletion events in cancer cells. In the spectral analysis plot, this kind of fluctuation in read density will often appear as a local maximum. For example, in Figure 5a we have a sample with a large number of duplicate reads. Note the spike in percentage variance that occurs at a length scale 2 kbp, indicating a large number of 'point spikes' in the density plot that rise and fall over 2 kbp intervals. This fluctuation disappears after de-duplicating reads, as shown in Figure 5b, suggesting that spectral analysis provides an efficient way of detecting PCR amplification bias during library preparation. The spectral analysis was done by using a decimated Haar wavelet decomposition, as described in [1].

The second component is a Poisson simulation. The idea is to perform a spectral analysis on an idealized set of tag counts that is unbiased, but is none the less sampled to the same depth (the same genome-wide mean tag count) and distribution of coverage (the same genome-wide spread in tag count). The spectral energy landscape of a sample with minimal bias will be similar to that of the simulation (compare Figure 5a and Figure 5c). To generate an unbiased simulation, we used a Poisson-Gamma mixture model. We performed the simulation by fitting a Gamma distribution to the set of tag counts per 1 kbp observed in the Input channel, using maximum likelihood. We then generated a list of tag counts by first sampling from the Gamma distribution and using this value as the mean of Poisson distribution. We then sampled from the Poisson distribution to obtain the tag count.

### Normalizing multiple IPs for differential analysis

For multiple IP differential analysis, CHANCE first normalizes each sample to the mean read depth over all samples considered. CHANCE then forms a consensus sample using a multi-channel signal combiner described in [17-19]. Briefly, given *n *IP samples, alignments are first binned into 1 kbp non-overlapping windows. Then, if *s_ij _*is the count in the *j*-th bin of the *i*-th sample, the combiner chooses positive weights {*w*_1_,...,*w*_n_} to form the consensus:

$$c_{j}=\overset{n}{\underset{i=1}{\sum }}w_{i}s_{ij}$$

The weights are chosen to maximize $\overset{n}{\underset{k=1}{\sum }}\overset{n}{\underset{l=1}{\sum }}M_{kl}w_{k}w_{l}$ such that $\overset{n}{\underset{i=1}{\sum }}w_{i}=1$, where *M_kl _*is the sample covariance matrix of *s_ij_*. See [17-19] for the derivation. This has the effect of determining a consensus whose background component will be the largest possible subset of the genome of mutual background for all *n *original samples. Lastly, SES is used to determine differential enrichment of each sample from the consensus, as well as the pairwise differential comparisons between samples.

### Spot validation

The user can provide CHANCE with a list of genomic loci to spot validate positive and negative control regions, such as those used in ChIP-qPCR prior to sequencing. The fold-change in tag count is reported. The reported *P*-value for each region is the probability of the tag count in the IP channel, under a Poisson null model with a mean equal to the observed tag count in the Input channel. This is not intended for peak calling but rather for validation and confirmation of CHANCE's other quality metrics. In other words, although a large fold-change and small Poisson *P*-value do not necessarily imply a successful IP, lack of enrichment in multiple positive control loci will suggest problems with sequencing.

### Comparison with ENCODE

The ENCODE project provides representative transcriptional and epigenetic maps of the mammalian genomes. We thus reasoned that the ENCODE data can provide a rough landscape of TF binding and epigenetic modification sites that are applicable to multiple cell types. The 'Comparison with ENCODE' module thus allows one to compare one's own dataset with corresponding ENCODE datasets to determine if the user's data show an accumulation of reads within ENCODE peaks. For each TF or epigenetic mark for which ENCODE has called peaks (Additional file 2), we assembled a union peak set. The union peak set is the union of all peaks for the same TF or histone mark from multiple cell types. We then count the fraction *p *of user reads that map to the union set in the IP channel, and the fraction *q *of reads that map to the union set from the Input channel. The relative odds of observing a read from the IP channel in the union set, compared to Input, can then be expressed by the odds ratio *p*/(1 - *p*)/*q*/(1 - *q*). We then compute the same odds ratio for each IP-Input pair, in ENCODE, for the same TF or histone mark. The distribution of odds ratios gives the user a sense of how cell type-specific enrichment for that particular mark is. If the user's odds ratio is much less than one, this indicates that the user's data set is somewhat of an outlier, compared to ENCODE. We compute the log of the odds ratio, since the log odds is approximately normal. This allows us to fit a normal curve to the distribution of ENCODE log odds ratios. The cumulative distribution at the log odds of the user's data then gives a probability indicating how much of an outlier the user's data set is. Although not definitive of a failed experiment on its own, a small odds ratio provides additional evidence of a potentially failed experiment.

### Software availability

CHANCE is open source, published under the GNU General Public License. The Matlab source code, User Guide, examples, and executables for Mac OS, Windows, and Linux are available at https://github.com/songlab/chance.

## Abbreviations

ChIP: chromatin immunoprecipitation; CHANCE: CHip-seq ANalytics and Confidence Estimation; GEO: Gene Expression Omnibus; GUI: graphical user interface; HESC: human embryonic stem cell; IP: immunoprecipitation; NSC: neural stem cell; qPCR: quantitative polymerase chain reaction; SES: signal extraction scaling; TF: transcription factor; UCSF: University of California: San Francisco.

## Authors' contributions

AD and JSS designed the project and developed the underlying algorithms. AD designed and developed the software package. AN implemented the BAM/SAM file reader interface. AD and AN wrote the User Guide, and all authors together wrote the paper.

## Supplementary Material

Additional file 1 **CHANCE feature comparison table**. Click here for file

Additional file 2 **CHANCE training data table**. This file enumerates the experiment information and url for each ENCODE sample used in either the training data set for the false discovery rate computation in the "IP enrichment" module or the "Comparison with ENCODE module". Click here for file
