# Supplementary material for: CHANCE: comprehensive software for quality control and validation of ChIP-seq data
Source: Genome Biol. 2012 Oct 15;13(10):R98. doi: 10.1186/gb-2012-13-10-r98 (PMC4053734; doi:10.1186/gb-2012-13-10-r98)
Supplement: Additional file 1 — CHANCE feature comparison table. [file gb-2012-13-10-r98-S1.PDF]

Additional File 1: CHANCE feature comparison table

Feature	CHANCE	BEADS[i]	htSeqTools[ii]	Avardis[iii]	FastQC[iv]	SAMStat[v]	Homer[vi]	Solexa QA[vii]	ConDeTri[viii]	CANGS[ix] FASTA	Galaxy[x]
File type support	BAM, SAM, BOWTIE, BED, tagAlign, MATLAB	GFF	none (R data structure)	BAM, SAM, BED, ELAND	BAM, SAM, FASTQ	SAM	BOWTIE, BED, ELAND	FASTQ	FASTQ		BAM, SAM, BED, WIG, Others
Does not require knowledge of programming/shell commands	X			X	X						X
Detects base call quality bias	X			X	X	X		X	X	X	X
Detects nucleotide content bias	X	X		X	X	X	X	X			X
Graphical interface	X			X	X						X
Coverage estimate	X		X				X				X
Classifies failed experiments and identifies reasons for failure	X										
Detects insufficient sequencing depth	X										
Detects batch effects	X										
Detects PCR amplification bias	X										
Does normalization of multiple (IP-Input) pairs	X										
Compares IP enrichment between multiple (IP-Input) pairs	X										
Compares overlap in signal between user data and a consensus of ENCODE samples	X										
Contains a full suite of plot tools	X										

[i] Cheung MS, Down Ta, Latorre I, Ahringer J: Systematic bias in high-throughput sequencing data and its correction by BEADS. Nucleic acids research 2011, 39(15), [http://www.ncbi.nlm.nih.gov/pubmed/21646344].

[ii] Planet E, Attolini CSO, Reina O, Flores O, Rossell D: htSeqTools: high-throughput sequencing quality control, processing and visualization in R. Bioinformatics (Oxford, England) 2012, 28(4):589 [http://www.ncbi.nlm.nih.gov/pubmed/22199381].

[iii] Avardis NGS [http://www.avadis-ngs.com/].

[iv] FastQC [http://www.bioinformatics.babraham.ac.uk/projects/fastqc/].

[v] Lassmann T, Hayashizaki Y, Daub CO: SAMStat: monitoring biases in next generation sequencing data. Bioinformatics (Oxford, England) 2011, 27:130

[http://www.pubmedcentral.nih.gov/articlerender.fcgi?artid=3008642n&tool=pmcentrezn&rendertype=abstract].

[vi] Homer [http://biowhat.ucsd.edu/homer/ngs/index.html].

[vii] Solexa QA [http://solexaqa.sourceforge.net/].

[viii] Smeds L, Kunstner A: ConDeTri - A Content Dependent Read Trimmer for Illumina Data. PLoS ONE 2011, 6(10):e26314, [http://dx.doi.org/10.1371n%2Fjournal.pone.0026314].

[ix] Pandey RV, Nolte V, Schlotterer C: CANGS: a user-friendly utility for processing and analyzing 454 GS-FLX data in biodiversity studies. BMC research notes 2010, 3:3, [http://www.pubmedcentral.nih.gov/articlerender.fcgi?artid=2830946n&tool=pmcentrezn&rendertype=abstract].

[x] Giardine B, Riemer C, Hardison RC, Burhans R, Elnitski L, Shah P, Zhang Y, Blankenberg D, Albert I, Taylor J, Miller W, Kent WJ, Nekrutenko A: Galaxy: a platform for interactive large-scale genome analysis. Genome research 2005, 15(10):1451 [http://www.pubmedcentral.nih.gov/articlerender.fcgi?artid=1240089n&tool=pmcentrezn&rendertype=abstract].
